# On the properties of the toxicity index and its statistical efficiency

**DOI:** 10.1002/sim.8858

**Published:** 2020-12-20

**Authors:** Zahra S. Razaee, Arash A. Amini, Márcio A. Diniz, Mourad Tighiouart, Greg Yothers, André Rogatko

**Affiliations:** ^1^ Biostatistics and Bioinformatics Research Center Cedars‐Sinai Medical Center Los Angeles California USA; ^2^ Department of Statistics University of California Los Angeles California USA; ^3^ University of Pittsburgh and NRG Oncology Pittsburgh Pennsylvania USA

**Keywords:** adverse events, Poisson‐Limit model, toxicity index, T‐rank preservation, two‐sample test

## Abstract

Cancer clinical trials typically generate detailed patient toxicity data. The most common measure used to summarize patient toxicity is the maximum grade among all toxicities and it does not fully represent the toxicity burden experienced by patients. In this article, we study the mathematical and statistical properties of the toxicity index (TI), in an effort to address this deficiency. We introduce a total ordering, (T‐rank), that allows us to fully rank the patients according to how frequently they exhibit toxicities, and show that TI is the only measure that preserves the T‐rank among its competitors. Moreover, we propose a Poisson‐Limit model for sparse toxicity data. Under this model, we develop a general two‐sample test, which can be applied to any summary measure for detecting differences among two population of toxicity data. We derive the asymptotic power function of this class as well as the asymptotic relative efficiency (ARE) of the members of the class. We evaluate the ARE formula empirically and show that if the data are drawn from a random Poisson‐Limit model, the TI is more efficient, with high probability, than the maximum and the average summary measures. Finally, we evaluate our method on clinical trial toxicity data and show that TI has a higher power in detecting the differences in toxicity profile among treatments. The results of this article can be applied beyond toxicity modeling, to any problem where one observes a sparse array of scores on subjects and a ranking based on extreme scores is desirable.

## 1 INTRODUCTION

Adverse event reporting has become a crucial step in the assessment of therapies in clinical trials since the first recommendation for grading toxicities was presented, following the efforts of the World Health Organization to standardize cancer treatment reports in the late 1970s.^1^, ^2^ An adverse event (AE), or a toxicity, is any unfavorable and unintended sign, symptom, or disease temporally associated with the use of a medical treatment or procedure that may or may not be considered related to the medical treatment or procedure.^3^ The National Cancer Institute (NCI) published the common toxicity criteria (CTC) in 1983, providing a standardized list of AE terms commonly encountered in oncology to guide investigators in identifying and documenting toxicities.^4^, ^5^

The CTC has evolved over the last decades to the current and wide‐spread common terminology criteria for adverse events,^3^ which is organized in 26 system organ classes, such that each AE term is defined and classified into grades of severity denoted as 0, 1, … , 5 with 0 corresponding to no symptoms, 1 corresponding to a mild symptom, up to 5 indicating death. While CTCAE allows investigators to assess and document toxicities in a systematized manner, it also creates a challenge in summarizing a large amount of data at the patient level. Each patient can experience more than one AE term from different organ systems and different grades of the same AE term during several cycles of treatment resulting in a vector of integers, the toxicity profile, of varying length containing all toxicities grades per patient. In recognition of deficiencies in toxicity reporting, NCI launched a Cancer Moonshot funding opportunity^6^ to accelerate research on improved approaches to evaluating the tolerability of cancer treatments.

Currently, the most used approach in phase III trials to summarize multiple toxicities per patient is the maximum‐grade (max‐grade).^7^, ^8^, ^9^ Other approaches such as those of References 10, 11, 12 have been proposed in the literature to address the lack of representation of the toxicity burden experienced by patients when the max‐grade method is applied. In particular, a longitudinal approach (Tox‐T)^12^ incorporating low‐grade toxicities and their duration using the average as a summary measure was introduced. Even though Tox‐T better represents the toxicity burden, the average grade is hard to interpret clinically by investigators. In the setting of dose finding in early phase cancer trials, many authors proposed statistical models and dose escalation designs that take into account all grades and types of toxicities with the goal of improving the safety and efficiency of the trial.^13^, ^14^, ^15^, ^16^, ^17^, ^18^ Some of these methods use multivariate models for characterizing the relationship between different grades of toxicities and dose while others, such as the Q‐TWIST,^19^ summarize the information from all toxicity grades into a continuous score. In general, under some scenarios for the location of the true maximum tolerated dose (MTD), a modest gain in the precision of the estimate of the MTD is achieved when including information from all toxicities relative to models that use a binary indicator of toxicity (a.k.a. dose limiting toxicity), without compromising the safety of the trial.

The *toxicity index (TI)*, proposed in Reference 20, is a summary measure that preserves the highest grade while incorporating lower grade toxicities. The TI can avoid the loss of information and improve clinical interpretability. It was recently shown that TI is more powerful than other common toxicity summaries in detecting differences among treatments^21^ in the National Surgical Adjuvant Breast and Bowel Project clinical trial (NSABP R0‐4).^22^, ^23^ To quantify the information loss, power curves were empirically estimated by resampling the available data, showing the relative performance of different toxicity summaries in comparing treatments.

While the TI was introduced more than a decade ago, its mathematical and statistical properties have never been studied. In this article, we report on the novel characteristics of TI as a summary measure and its effectiveness in comparing treatments. We also develop a framework for modeling and ranking toxicity data, which as discussed below, is applicable beyond drug toxicity problems. Our contributions in this article are as follows:

1. We propose a framework for modeling the data in experiments where scores on multiple events are recorded for a collection of subjects. In particular, the model is applicable to *n* × *d* arrays of scores on *n* subject and *d* adverse events, where each entry is a score in {0, 1, … , *K*}. We refer to the elements of the latter set as *grades*. We consider *sparse* arrays where most of the scores are zero, and argue in favor of a Poisson‐Limit model that simplifies downstream analysis. The model can be equivalently expressed in terms of the *reduced frequency vectors (RFV)* of the subjects where, for each subject, one records the frequency of each observed grade except zero.
2. We introduce a total ordering, referred to as the *T*‐order, on the space of RFVs, that allows for comparing them based on how frequently they exhibit extreme grades. Since the *T*‐order is a total order (ie, every pair of vectors are comparable), it induces a full ranking among RFVs which we call the *T*‐rank. This, for example, allows one to fully rank subjects (or treatments), strictly and without ties, except when the RFV vectors are exactly equal. The *T*‐rank, in particular, is relevant to drug toxicity trials where one wants to emphasize differences in extreme toxicity. The ranking is also useful in any application where variations in the extreme scores among subjects or treatments are of concern.
3. We consider two‐sample testing based on score data coming from two different Poisson‐Limit models. We propose a general test that compares the two treatments by looking at the difference in mean between values of a summary measure *g*(·) applied to each subject in each treatment. We study three candidates for *g*(·): the TI, the average, and the maximum score. We derive exact expressions for the mean and variance of the test statistic under the Poisson‐Limit model, allowing us to analytically set the critical region of the test with guarantees on the asymptotic significance level.
4. Our result also provides an approximate analytical formula for the power function for each test, as well as an exact expression for the slope of the test as defined by Vaart^24^ ^(chapter 14)^. The slopes allow us to analytically calculate the asymptotic relative efficiency (ARE) of the three summary measures with respect to each other. We evaluate these ARE expressions empirically, showing that in the majority of the cases, TI is more efficient than the maximum and much more efficient than the average.
5. We demonstrate our theoretical results on the two‐sample test by fitting the Poisson‐Limit model to real data from a clinical trial and evaluating the power function for tests based on the three summary measures. The resulting plots complement those of Reference 21 which are obtained based on resampling the data, and confirm the superiority of TI in detecting the differences in toxicity profile among drugs.

The organization of the rest of the article is as follows: Section 2 provides background on the toxicity data and the summary measures. We then introduce the Poisson‐Limit model for such data. In Section 3, we derive mathematical properties of TI, including monotonicity with respect to the *T*‐rank. Deficiencies of competing summary measures in preserving the ranks are also discussed here. Section 4 develops our two‐sample test and presents analytical results on the power function and test slopes. The methodology is illustrated with simulations in Section 5 and conclude with a discussion in Section 7.

*Notation*. We use ℤ for the set of integers, ${\mathbb{R}}_{+}$ for the set of nonnegative real numbers, and ${\mathbb{Z}}_{+}$ for the set of nonnegative integers. We write [*K*] = {1, … , *K*} and [*K*]_∗_ = {0, 1, … , *K*}. The indicator of a set *A* is denoted as 1{*x* ∈ *A*}, evaluating to 1 if *x* ∈ *A* and 0 otherwise.

## 2 DATA, MODELS, AND SUMMARY MEASURES

Assume that we have a collection of *d* AEs that we represent as {1, … , *d*}. For each subject in a given period of time (one cycle or multiple cycles combined), we observe a toxicity profile that can be viewed as a vector $Y=(Y_{1},Y_{2},\ldots ,Y_{d})\in {\mathbb{Z}}_{+}^{d}$, where *Y*_*i*_ represents the toxicity grade of AE *i*. Generally, we assume that there is a maximum possible grade *K*, so that we can assume $Y\in {\left[K\right]}_{*}^{d}$, where we recall that [*K*]_∗_ = {0, 1 … , *K*}. We assume that the corresponding grades for different AEs are equivalent, that is, grade 1 in *Y*_1_ is equivalent to grade 1 in *Y*_2_, and so on. The toxicity index is an example of a summary measure that maps *Y* to a scalar grade. More precisely, let *Y*_(1)_ ≥ *Y*_(2)_ ≥ ⋯ ≥ *Y*_(*d*)_ be the order statistic of *Y*. The toxicity index was originally defined as a function $\tau :{\mathbb{Z}}_{+}^{d}\to {\mathbb{R}}_{+}$ given by:^20^

(1) $$\begin{array}{ll} \tau (Y):=\overset{d}{\underset{i=1}{\sum }}\frac{Y_{\left(i\right)}}{w_{i}(Y)},\;\text{where}\;w_{i}(Y):=\overset{i-1}{\underset{j=1}{\prod }}(1+Y_{\left(j\right)}). & \end{array}$$

Besides the toxicity index, there are other summary measures, which are often defined in terms of the frequency vector of the grades, that is, *X*_∗_ = (*X*_0_, *X*_1_, *X*_2_, … , *X*_*K*_) where $X_{r}=\sum_{j=1}^{d}1\{Y_{j}=r\}$. Since we are mainly interested in sparse toxicity profiles, level 0 has a special status. We will work with the reduced frequency vector *X* = (*X*_1_, … , *X*_*K*_) that only retains the frequency of nonzero levels. Let us write $X_{+}=\sum_{r=1}^{K}X_{r}$ and note that *X*_0_ = *d* − *X*_+_, that is, there is no loss of information working with *X* instead of *X*_∗_.

Common summary measures can be stated in terms of the reduced frequency vector *X*. The following two examples are of particular interest:

1. The mean index which can be represented as
  (2) $$\begin{array}{ll} \text{avg}(X)=\frac{\sum_{r=1}^{K}rX_{r}}{\sum_{r=1}^{K}X_{r}}1\{X_{+}>0\}. & \end{array}$$
2. The maximum index which is given by
  (3) $$\begin{array}{ll} \text{mx}(X)=\max\{r\in [K]:X_{r}>0\}, & \end{array}$$
  where we interpret the maximum of the empty set as 0.

Throughout, maximum index, mx, and max‐grade will be used interchangeably.

### 2.1 The Poisson‐Limit model

To study the statistical properties of the summary measures, we propose a model for the toxicity data. We start with a model, where each entry of *Y* is an i.i.d. draw from a categorical variable with levels in [*K*]_∗_. That is, {*Y*_*j*_} are i.i.d., with $\mathbb{P}(Y_{j}=r)=p_{r}$ for *r* ∈ [*K*]_∗_ and *j* ∈ [*d*]. In the absence of prior knowledge about toxicities, the i.i.d. assumption is a reasonable first approximation. It follows that the frequency vector *X*_∗_ has a multinomial distribution with parameter *d* and *p*_∗_ = (*p*_0_, *p*_1_, … , *p*_*K*_). Formally, *X*_∗_ ∼ Mult(*d*, *p*_∗_).

Under the above model, we denote the distribution of the reduced frequency vector *X*, as Mult_|0⟨_(*d*, *p*) where *p* = (*p*_1_, … , *p*_*K*_). In other words, *X* ∼ Mult_|0⟨_(*d*, *p*) if

$$\left(d-X_{+},X\right)∼\text{Mult}\left(d,\left(1-p_{+},p\right)\right),$$

where $X_{+}=\sum_{r=1}^{K}X_{r}$ and $p_{+}=\sum_{r=1}^{K}p_{r}$. We are interested in the cases where the toxicity profile is sparse, that is, many of {*Y*_*j*_} are often zero. In those cases, it is reasonable to assume that $p_{r}=\lambda_{r}/d$ for *r* ∈ [*K*]. Letting $\lambda =(\lambda_{1},\ldots ,\lambda_{K})$, our model is equivalent to

(4) $$\begin{array}{ll} X∼{\text{Mult}}_{|0\langle }\left(d,\frac{\lambda }{d}\right). & \end{array}$$

As shown in the Appendix (Section A0.1), *X* converges in distribution to a product of Poisson distributions, that is,

(5) $$\begin{array}{ll} X⇝\text{Poi}(\lambda ):=\overset{K}{\underset{r=1}{\prod }}\text{Poi}(\lambda_{r}),\;d\to \infty . & \end{array}$$

We will use this limiting distribution in our statistical analysis. It is a good approximation for large sparse toxicity profiles and allows us to derive explicit analytical expressions for various statistical quantities of interest. We refer to (5) as the *Poisson‐Limit model*.

Remark 1 Even when the distributions of the AEs are allowed to be different, that is, $\mathbb{P}(Y_{j}=r)=q_{jr}$ with *q*_*jr*_ potentially varying with *j*, we still have a Poisson‐Binomial distribution for each marginal (ie, the distribution of $\sum_{j=1}^{d}1\{Y_{j}=r\}$ for a given *r*), which can be approximated by a Poisson distribution in the sparse case.

## 3 TOXICITY INDEX PRESERVES *T*‐rank

In this section, We derive some analytical properties of the toxicity index. We first show how the toxicity index can be computed based only on the reduced frequency vector (RFV) of the observations. We define a total ordering, referred to as the *T*‐rank, on the space of RFVs and show that the toxicity index preserves this ordering in a strict sense. A consequence of this result is that the toxicity index is a one‐to‐one mapping on the RFV space, hence there is no loss of information when summarizing the toxicity profile this way.

### 3.1 Closed‐form representation

Recall that $Y\in {\left[K\right]}_{*}^{d}$ represents the toxicity profile of a patient. Consider the truncated toxicity index:

$$\tau_{k}(Y):=\overset{d}{\underset{i=1}{\sum }}\frac{Y_{\left(i\right)}}{w_{i}(Y)}1\{Y_{\left(i\right)}\leq k\}$$

which only considers toxicities of grades *k* or lower. We have $\tau (Y)=\tau_{K}(Y)$ where *K* is the maximum toxicity grade observed in *Y*.

Let $x=x(Y)\in {\mathbb{R}}^{K}$ be the reduced frequency vector of *Y*: $x_{k}=x_{k}(Y)=\sum_{i=1}^{n}1\{Y_{i}=k\}$ for all *k* = 1, … , *K*. The following proposition provides a recursive formula for $\tau_{k}(\cdot )$ in terms of *x*:

Proposition 1 *For any* $x\in {\mathbb{R}}^{K}$ *, let* $g_{k}(x)=\prod_{i=k}^{K}{\left(1+i\right)}^{-x_{i}}$ *for* *k* = 2, … , *K* *and* *g*_*K* + 1_(*x*) = 1 *. We have, for* *k* = 1, … , *K* *,*

$$\tau_{k}(Y)-\tau_{k-1}(Y)=(k+1)g_{k+1}(x)[1-{\left(1+k\right)}^{-x_{k}}],$$

*where* *x* = *x*(*Y*) *is the reduced frequency vector of Y and* $\tau_{0}(\cdot ):=0$.

It follows from Proposition 1 that the toxicity index τ, defined in (1), has the following closed form in terms of the frequency vector: τ(Y)=TI(x(Y)) where

(6) $$\begin{array}{ll} \text{TI}(x):=\overset{K+1}{\underset{k=2}{\sum }}kg_{k}(x)\left(1-k^{-x_{k-1}}\right). & \end{array}$$

Letting ${\bar{g}}_{k}(x):=1-g_{k}(x)$, we can write TI(·) alternatively as follows:

$$\text{TI}(x)=\overset{K+1}{\underset{k=2}{\sum }}k[g_{k}(x)-g_{k-1}(x)]=2{\bar{g}}_{1}(x)+\overset{K}{\underset{k=2}{\sum }}{\bar{g}}_{k}(x).$$

For *K* = 5, with *x* = (*x*_1_, *x*_2_, *x*_3_, *x*_4_, *x*_5_), one can also rewrite (6) as

(7) $$\begin{array}{ll} \text{TI}(x)=6-6^{-x_{5}}(1+5^{-x_{4}})-2^{-2x_{3}-x_{5}}3^{-x_{2}-x_{5}}5^{-x_{4}}\left(1+3^{x_{2}}+2^{1-x_{1}}\right) & \end{array}$$

which could be faster for computations. In the sequel, we also refer to TI(·) as the toxicity index, based on the equivalence just established.

### 3.2 Monotonicity and T‐rank preservation

We now introduce a total order on the space of reduced frequency vectors (RFVs). In fact, the total order can be defined over all real‐valued *K*‐dimensional vectors:

Definition 1 (T‐order) For $x,y\in {\mathbb{R}}^{K}$, we write *x* ≻ *y* or *y* ≺ *x* if *x* ≠ *y* and

$$x_{i(x,y)}>y_{i(x,y)},\;\text{where}\;i(x,y):=\max\{i:x_{i}\neq y_{i}\}.$$

In words, for any two vectors *x* and *y* that are not equal, we let *i*(*x*, *y*) be the largest grade (last position) on which they differ and say that *x* ≻ *y* if the coordinate of *x* at *i*(*x*, *y*) is larger than the corresponding coordinate of *y*. We write *x* ≽ *y* if either *x* = *y* or *x* ≻ *y*, and similarly for *x* ≼ *y*.

The T‐order, defined by ≽, is a total order on ${\mathbb{R}}^{K}$, that is, we can compare any pair of vectors in ${\mathbb{R}}^{K}$. To see this, it is enough to verify the transitivity property:

Lemma 1 (T‐order is transitive) *If* *x* ≻ *y* *and* *y* ≻ *z* *, then* *x* ≻ *z*.

Recall that the toxicity index can be written as a function TI(·) of the reduced frequency vector; see (6). The toxicity index TI(·) is (strictly) increasing on ${\mathbb{Z}}_{+}^{K}$, with respect to the T‐order:

Theorem 1 *Let* *K* ≥ 2 *. For any* $x,y\in {\mathbb{Z}}_{+}^{K}$ *, if* *x* ≻ *y* *, then* TI(*x*) > TI(*y*).

**FIGURE 1:**
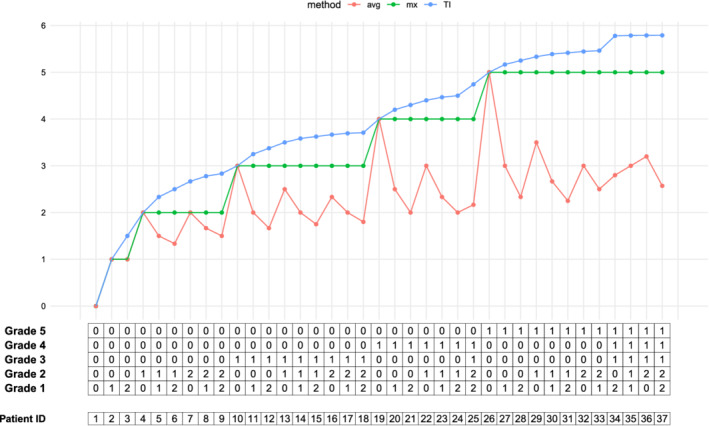
TI preserves the T‐rank while the mx and avg do not. The *x*‐axis is an array, with 37 patients with each column representing a RVF for an individual, ordered in increasing *T*‐rank. Each cell in the array is the number of AEs experienced at a particular grade for a given cycle of treatment. The TI exhibits strict monotonicity with respect to this ordering while the mx is nondecreasing and avg is neither monotone nor nondecreasing [Colour figure can be viewed at wileyonlinelibrary.com]

Since the T‐order is a total order, it provides a ranking of all elements of ${\mathbb{Z}}_{+}^{K}$. We refer to this ranking as the *T‐rank*. Theorem 1 shows that the toxicity index preserves the T‐rank. Theorem 1 also shows that TI(·) is injective (ie, one‐to‐one) on ${\mathbb{Z}}_{+}^{K}$. The significance of the *T*‐order, and the associated *T*‐rank, is that it is sensitive to the highest grade on which the two RFVs *x* and *y* differ. This is the desired way to compare RFVs derived from toxicity profiles, since the highest grade toxicities are the most important. The two other common summary measures, namely, mx(·) and avg(·) do not preserve the *T*‐rank:

The max‐grade mx(*x*) is a nondecreasing function w.r.t. the T‐rank but is not one‐to‐one, that is, we can have *x* ≻ *y* but *g*(*x*) = *g*(*y*). The average function avg(*x*) is neither monotone w.r.t. to the T‐rank nor one‐to‐one. For example, consider *K* = 2, and take *x* = (6, 2) and *y* = (2, 1). Then, *x* ≻ *y* but avg(*x*) = 1.25 < avg(*y*) = 1.33. It is also easy to find cases where *x* ≻ *y* and avg(*x*) > avg(*y*) or avg(*x*) = avg(*y*), for example, take *x* = (2, 2) or *x* = (4, 2), respectively. Figure 1 illustrates the TI vs mx and avg as functions of some sorted vectors based on T‐rank showing that TI is both monotone and one‐to‐one unlike the mx and avg and thus preserves ranking.

Intuitively, the *T*‐rank is a natural way to order patients according to their toxicity grades. We look at the largest grade first, and if the frequency of that grade is higher in one patient we rank their toxicity higher. Otherwise (ie, in case of equality), we look at the second largest grade and compare the frequencies there and so on. As an example, in the hypothetical dataset, discussed in Section 6.1, the RFVs of patients 7 and 8 are *x* = (0, 1, 3, 1, 0) and *y* = (0, 4, 1, 1, 0), respectively. These two match on grades 5 and 4 but not 3. Then, the largest grade that they differ on is grade 3, that is, *i*(*x*, *y*) = 3. Since *x*_3_ > *y*_3_, we conclude that patient 7 has a higher *T*‐rank than patient 8. This way we can rank all patients. The mx function when plotted against this order looks like a step function while TI always monotonically increases, as shown in Figure 1. The avg mixes the high and low grades which generally causes the overall grade to go down, hence misrepresenting the toxicity as determined by the *T*‐rank; this is shown in Figure 1 as average occasionally going down even when the toxicity rank goes up.

## 4 STATISTICAL THEORY

In this section, we consider the statistical problem of testing whether two populations of toxicity profiles are different. This is the central problem of pharmacology where one is interested in determining whether two or more drugs have the same toxicity effects in patients, or whether a certain drug is more toxic than the other. We present the problem under the Poisson‐Limit model of Section 2.1, and introduce a general two‐sample test that can be applied based on any scalar summary measure. We present an asymptotic analysis of these tests (Theorem 2) and compute the relevant parameters for the three summary measures: the average, the maximum, and the TI.

### 4.1 Two‐sample tests

Recall the Poisson‐Limit model of Section 2.1 for the reduced frequency vectors (RFVs). Assume that we observes RFV samples from two such populations:

(8) $$\begin{array}{ll} X^{\left(1\right)},\ldots ,X^{\left(n\right)}∼\text{Poi}(\lambda ),\;Y^{\left(1\right)},\ldots ,Y^{\left(m\right)}∼\text{Poi}(\gamma ), & \end{array}$$

where $\lambda =(\lambda_{r})$ and $\gamma =(\gamma_{r})$ are vectors in ${\mathbb{R}}_{+}^{K}$. We recall that $\text{Poi}(\lambda )=\prod_{r}\text{Poi}(\lambda_{r})$ is the distribution with independent $\text{Poi}(\lambda_{r})$ coordinates. Our goal is to test the null hypothesis $H_{0}:\lambda =\gamma$. We consider the general statistic

$$S_{g}=\frac{1}{n}\overset{n}{\underset{i=1}{\sum }}g(X^{\left(i\right)})-\frac{1}{m}\overset{m}{\underset{i=1}{\sum }}g(Y^{\left(i\right)}),$$

for some mapping *g* from ${\mathbb{R}}^{d}$ to ℝ. Let us define

(9) $$\begin{array}{ll} M_{g}(\lambda ):={𝔼}_{\lambda }[g(X)],\;\sigma_{g}^{2}(\lambda ):={\mathrm{var}}_{\lambda }(g(X)), & \end{array}$$

where ${𝔼}_{\lambda }$ and ${\mathrm{var}}_{\lambda }$ denote the expectation and variance, assuming that X∼Poi(λ). We assume that $M_{g}(\lambda )$ and $\sigma_{g}^{2}(\lambda )$ are finite for all $\lambda \in {\mathbb{R}}_{+}^{K}$. Consider the two‐sample test that rejects the null hypothesis if

(10) $$\begin{array}{ll} |S_{g}|>z_{\alpha /2}\sqrt{\sigma_{g}^{2}(\hat{\lambda })\left(\frac{1}{n}+\frac{1}{m}\right)}, & \end{array}$$

where $\hat{\lambda }$ is the following estimate

(11) $$\begin{array}{ll} \hat{\lambda }=\frac{1}{m+n}\left(\overset{n}{\underset{i=1}{\sum }}X^{\left(i\right)}+\overset{m}{\underset{j=1}{\sum }}Y^{\left(j\right)}\right). & \end{array}$$

The next theorem describes the asymptotic behavior of this test:

Theorem 2 *Let* ${\left\{X^{\left(i\right)}\right\}}_{i=1}^{n}$ *and* ${\left\{Y^{\left(i\right)}\right\}}_{i=1}^{m}$ *be generated from model (* *8* *), with some* $\lambda ,\gamma \in {\mathbb{R}}_{+}^{K}$ *. Assume that* *n*, *m* → *∞* *such that* n/m→ρ *and that* $\sigma_{g}^{2}(\cdot )$ *is a continuous function. Then, the two‐sample test that rejects the null according to (* *10* *), has asymptotic level* α *. Moreover*:

(a) *If* $M_{g}(\lambda )\neq M_{g}(\gamma )$ *, the test is asymptotically consistent (ie, the power converges to 1)*.
(b) *If* $M_{g}(\lambda )=M_{g}(\gamma )$ *, the test is inconsistent and its power converges to*
  $$2Q\left(z_{\alpha /2}\sqrt{\frac{1+\rho }{1+\rho \nu }}\right),$$
  *where* $\nu =\sigma_{g}^{2}(\gamma )/\sigma_{g}^{2}(\lambda )$ *. In particular, if in addition* $\sigma_{g}^{2}(\gamma )=\sigma_{g}^{2}(\lambda )$ *, the test is asymptotically powerless (ie, the power converges to* α *)*.
(c) *Assume that* *M*_*g*_(·) *is continuously differentiable at* λ *. For the shrinking alternative,* $\gamma =\lambda +\delta /\sqrt{n}$ *with* $\delta \in {\mathbb{R}}^{K}$ *, the power of the test converges to*
  (12) $$\begin{array}{ll} Q(z_{\alpha /2}+B)+Q(z_{\alpha /2}-B),\;\text{where}\;B=\frac{\left\langle \nabla M_{g}(\lambda ),\delta \right\rangle }{\sigma_{g}(\lambda )\sqrt{1+\rho }}. & \end{array}$$

The quantity *B*^2^, with *B* given in (12), is the slope of the test in the direction δ. The slope plays a role in determining the Pitman asymptotic relative efficiency (ARE) of two test with respect to each other. In particular, the ratio of the slopes for two tests determines the asymptotic ratio of the samples sizes necessary for achieving the same power by two tests of a given size; see Reference 24, chapter 14 for details. The proof of Theorem 2 also gives the following asymptotic approximation to the power of the test:

(13) $$\begin{array}{ll} \text{power}\approx Q\left(\frac{\tau +\sqrt{n}\mu }{\sigma }\right)+Q\left(\frac{\tau -\sqrt{n}\mu }{\sigma }\right),\;\text{for}\;n\gg 1, & \end{array}$$

where $\mu =M_{g}(\gamma )-M_{g}(\lambda )$, $\tau =z_{\alpha /2}\sigma_{g}(\lambda )\sqrt{1+\rho }$ and $\sigma^{2}=\sigma_{g}^{2}(\lambda )+\rho \sigma_{g}^{2}(\gamma )$. Simulations in Section 5 show that this approximate formula is quite accurate even for small *n*.

Remark 2 One can replace $\sigma_{g}^{2}(\hat{\lambda })$ in (10) with any consistent estimate of the variance of the pooled sample (under null). Theorem 2 still holds for such a test. In particular, we can replace $\sigma_{g}^{2}(\hat{\lambda })$ with the empirical variance of the pooled sample: ${\hat{\sigma }}^{2}:=\frac{1}{m+n-1}\left[\sum_{i=1}^{n}{\left(X^{\left(i\right)}-\hat{\lambda }\right)}^{2}+\sum_{j=1}^{m}{\left(Y^{\left(j\right)}-\hat{\lambda }\right)}^{2}\right]$. The resulting test will be the usual two‐sample *t*‐test and it enjoys the same asymptotic properties as those of (10). There could be some advantage in using $\sigma_{g}^{2}(\hat{\lambda })$ vs ${\hat{\sigma }}^{2}$ for small sample sizes, but it is generally hard to quantify the difference since both estimates quickly approach the true variance under the null. The main utility of computing the functions $\sigma_{g}^{2}(\cdot )$ and *M*_*g*_(·) is that they enable us to obtain explicit expressions for the asymptotic power of the tests and the AREs.

To implement the test (10), one needs the variance function $\sigma_{g}^{2}(\cdot )$. To compute the slope, we additionally need the mean function *M*_*g*_(·). Below we derive exact expressions for these quantities for the three summary statistics of Section 2, that is, *g* ∈ {TI, mx, avg}. To simplify the notation, let $\lambda_{+}=\sum_{r=1}^{K}\lambda_{r}$, ${\bar{\lambda }}_{r}=\lambda_{r}/\lambda_{+}$, and $\bar{\lambda }=\lambda /\lambda_{+}$. Note that $\bar{\lambda }$ is a probability distribution on [*K*]. Let us denote the first and second moments of this probability distribution as

(14) $$\begin{array}{ll} m_{1}(\bar{\lambda })=\underset{r}{\sum }r{\bar{\lambda }}_{r},\;m_{2}(\bar{\lambda })=\underset{r}{\sum }r^{2}{\bar{\lambda }}_{r}. & \end{array}$$

We also define the function

$$\text{Er}(\lambda ):=\int_{0}^{1}\frac{e^{\lambda t}-1}{t}dt=\text{Ei}(\lambda )-\log\lambda -\gamma ,$$

where Ei is the exponential integral and γ is the Euler‐Mascheroni constant.

Lemma 2 *Under a Poisson model,* X∼Poi(λ) *, we have*

$$\begin{array}{llll} M_{\text{avg}}(\lambda ) & =(1-e^{-\lambda_{+}})m_{1}(\bar{\lambda }),\; & & \\ \sigma_{\text{avg}}^{2}(\lambda ) & =e^{-\lambda_{+}}\left\{m_{1}^{2}(\bar{\lambda })(1-e^{-\lambda_{+}})+\text{Er}(\lambda_{+})\left[m_{2}(\bar{\lambda })-m_{1}^{2}(\bar{\lambda })\right]\right\}.\; & & \end{array}$$

Corollary 1 *Assume that* $\lambda_{+}=\gamma_{+}$ *and* $m_{1}(\bar{\lambda })=m_{1}(\bar{\gamma })$ *. Then, the test (* *10* *) based on* *g* = avg *is inconsistent. If in addition,* $m_{2}(\bar{\lambda })=m_{2}(\bar{\gamma })$ *, then the test is powerless*.

It is known that $0\leq \text{Er}(\lambda )-[1-(3\lambda /4)]\leq 11\lambda^{2}/36$ for λ≥0, hence $e^{-\lambda }\text{Er}(\lambda )\to 0$ as λ→∞. This implies that for sufficiently large $\lambda_{+}$, the variance $\sigma_{\text{avg}}(\lambda )$ is nearly completely determined by $m_{1}(\bar{\lambda })$. Thus equality of the first moments of $\bar{\lambda }$ and $\bar{\gamma }$ together with $\lambda_{+}=\gamma_{+}\gg 1$ is enough for the mean index test to be almost powerless. Corollary 1, in fact, suggests that rather that the null hypothesis of $H_{0}:\lambda =\gamma$, the test based on avg is appropriate for testing the following null:

(15) $$\begin{array}{ll} H_{0}:\lambda_{+}=\gamma_{+},\;m_{r}(\bar{\lambda })=m_{r}(\bar{\gamma }),\;\text{for}\;r=1,2. & \end{array}$$

Remark 3 A counterintuitive consequence of Lemma 2 is that there are examples of rate vectors λ and γ, such that λ≥γ coordinatewise, but $M_{\text{avg}}(\lambda )<M_{\text{avg}}(\gamma )$. See Section 6.1 for such an example.

Next, we consider the maximum index: For *z* = (*z*_1_, … , *z*_*K*_, *z*_*K* + 1_), with *z*_*K* + 1_ = 1, define

(16) $$\begin{array}{ll} \xi (z):=\overset{K}{\underset{k=1}{\sum }}k\left[e^{-z_{k+1}}-e^{-z_{k}}\right]=\overset{K}{\underset{i=1}{\sum }}(1-e^{-z_{i}}). & \end{array}$$

See Section A0.3 for a derivation of the second equality.

Lemma 3 *Under a Poisson model,* X∼Poi(λ) *, we have*

$$\begin{array}{llll} M_{\text{mx}}(\lambda ) & =\xi \left(W_{*}(\lambda )\right),\; & & \\ \sigma_{\text{mx}}^{2}(\lambda ) & =\overset{K}{\underset{k=1}{\sum }}k^{2}\left[e^{-W_{k+1}(\lambda )}-e^{-W_{k}(\lambda )}\right]-M_{\text{mx}}^{2}(\lambda ),\; & & \end{array}$$

*where* $W_{*}(\lambda )={\left(W_{k}(\lambda )\right)}_{k=1}^{K+1}$ *with* $W_{k}(\lambda )=\sum_{r=k}^{K}\lambda_{r}$.

Finally, we consider the TI index. Let *a*_*i*_ = *i*/(*i* + 1) and *b*_*i*_ = *i*(*i* + 2)/(*i* + 1)^2^, and define

$$\begin{array}{ll} U_{k}(\lambda ):=\overset{K}{\underset{i=k}{\sum }}a_{i}\lambda_{i},\;V_{k,\ell }(\lambda ):=\overset{k\vee \ell -1}{\underset{i=k\wedge \ell }{\sum }}a_{i}\lambda_{i}+\overset{K}{\underset{i=k\vee \ell }{\sum }}b_{i}\lambda_{i}. & \end{array}$$

A sum with lower limit higher than the upper limit evaluates to zero, e.g., $U_{K+1}(\lambda )=0$. Let $U_{*}(\lambda )={\left(U_{k}(\lambda )\right)}_{k=1}^{K+1}$. We also define

(17) $$\begin{array}{ll} \Gamma_{k,\ell }(\lambda ):=e^{-V_{k,\ell }(\lambda )}-e^{-[U_{k}(\lambda )+U_{\ell }(\lambda )]}. & \end{array}$$

Lemma 4 *Under a Poisson model,* X∼Poi(λ) *, we have*

(18) $$\begin{array}{ll} M_{\text{TI}}(\lambda ) & =[1-e^{-U_{1}(\lambda )}]+\xi \left(U_{*}(\lambda )\right), \end{array}$$

(19) $$\begin{array}{ll} \sigma_{\text{TI}}^{2}(\lambda ) & =\overset{K+1}{\underset{k=2}{\sum }}\overset{K+1}{\underset{\ell =2}{\sum }}k\ell \left[\Gamma_{k,\ell }(\lambda )-\Gamma_{k-1,\ell }(\lambda )-\Gamma_{k,\ell -1}(\lambda )+\Gamma_{k-1,\ell -1}(\lambda )\right]. \end{array}$$

### 4.2 Computing the slope

The slope of the test (10) is *B*^2^, where *B* is defined in (12). To compute the slope, we need the gradient of the mean function. Let us consider the case of mx. The mean function is of the form $M_{\text{mx}}(\lambda )=\sum_{i=1}^{K}\psi ({\left[W\lambda \right]}_{i})$ where $\psi (z)=1-e^{-z}$ and *W* is a *K* × *K* upper triangular matrix with *i*th row $w_{i}^{T}=(w_{ij})$ such that *w*_*ij*_ = 1 for *j* ≥ *i*. It follows that

$$\nabla M_{\text{mx}}(\lambda )=\overset{K}{\underset{i=1}{\sum }}\;w_{i}\psi^{\prime }(w_{i}^{T}\lambda )=W^{T}\psi^{\prime }(W\lambda ),$$

where $\psi^{\prime }(z)=e^{-z}$ is applied coordinatewise to vector Wλ. The mean function for the TI statistic has a similar form: $M_{f}(\lambda )=2\psi ({\left[U\lambda \right]}_{1})+\sum_{i=2}^{K}\psi ({\left[U\lambda \right]}_{i})$, where *U* = (*u*_*ij*_) is an upper triangular matrix with *u*_*ij*_ = *j*/(*j* + 1) for *j* ≥ *i*. The rest of the calculations follow similarly. For the avg, the *i*th element of the gradient of the mean function is

$$\frac{\partial M_{\text{avg}}(\lambda )}{\partial \lambda_{i}}=e^{-\lambda_{+}}m_{1}(\bar{\lambda })-\frac{i}{{\bar{\lambda }}^{2}}(1-e^{-\lambda_{+}}).$$

We will use these expressions to empirically evaluate the slopes of the three summary measures, and compare their asymptotic relative efficiencies.

## 5 SIMULATIONS

We start by investigating the asymptotic relative efficiency (ARE) of TI w.r.t. the mx and avg summary measures. To do so, we consider the two‐sample Poisson‐Limit model (8) with *K* = 5 and generate the mean vectors, λ and γ, randomly as follows: λ is drawn from $\prod_{i=1}^{K}\text{Unif}(0.01,2)$ and γ is set equal to λ+δ, with $\delta ∼\prod_{i=1}^{K}\text{Unif}(0.05,0.3)$. We generate 10^4^ such parameter pairs and for each model, evaluate the ratio of the test slopes for TI vs mx and avg as a measure of the ARE, that is, we calculate $B_{\text{TI}}^{2}/B_{\text{mx}}^{2}$ and $B_{\text{TI}}^{2}/B_{\text{avg}}^{2}$.

**FIGURE 2:**
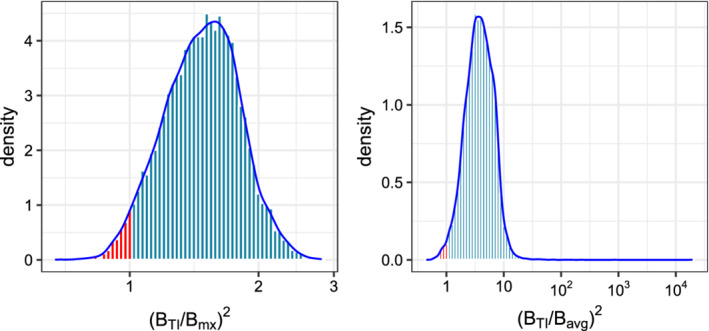
Comparison of the asymptotic relative efficiency, as the ratio of test slopes, for the TI vs mx (left), and TI vs avg (right). The plots are the histograms of the ARE under a random Poisson‐Limit model, described in Section 5. The portions colored red correspond to models where the ARE of TI is lower than the competitors [Colour figure can be viewed at wileyonlinelibrary.com]

Figure 2 illustrates the resulting histogram for the two slopes. Values above one indicate a higher efficiency for TI relative to the competing method. The plots show that, in the majority of cases, TI has a higher asymptotic efficiency compared to mx and avg. The histogram for $B_{\text{TI}}^{2}/B_{\text{avg}}^{2}$ has a heavier tail than $B_{\text{TI}}^{2}/B_{\text{mx}}^{2}$ which indicates that there are cases that TI achieves a much higher efficiency relative to avg (compared with mx). Under our sampling scheme, the probability that TI has a larger slope than the mx and the avg is 0.97 and 0.99, respectively.

**FIGURE 3:**
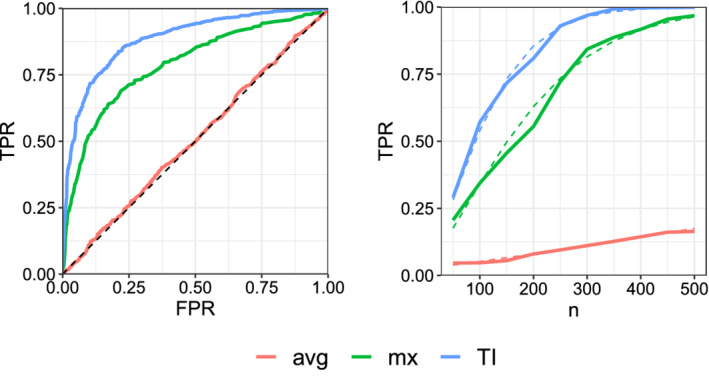
ROC curves (left) and the simulated and asymptotic power plots at significance level α=.05 (right). The dashed lines, in the right panel, denote the asymptotic power functions [Colour figure can be viewed at wileyonlinelibrary.com]

Next, we consider specific values for λ and γ, namely λ=[0.5,0.75,1,0.75,0.5] and γ=[0.60,1.05,1.50,1.05,0.60], and investigate the receiver operating characteristic (ROC) and the power curves for the three tests. The ROC is obtained by plotting the true positive rate (TPR) achievable for any given false positive rate (FPR). Note that TPR is an alternative name for the power; similarly, FPR is another name for the test size. The power curves are obtained by plotting the power of the test of size α=.05, against the sample size *n*. Figure 3 shows the ROC curves of the TI, mx and avg for *n* = 100 and also the asymptotic and simulated power plots for 1000 repetitions, illustrated with the dashed and solid lines, respectively. The values of λ and γ are chosen to be close to the setting of Corollary 1. In particular, we have $\lambda_{+}=3.5$, $\gamma_{+}=4.8$ and for the avg test, $m_{1}(\bar{\lambda })=m_{1}(\bar{\gamma })=3$, $m_{2}(\bar{\lambda })=10.57$, $m_{2}(\bar{\gamma })=10.44$. Corollary 1 explains the poor performance of the avg in this setting, matching what Figure 3 shows: The avg is nearly powerless for this testing problem.

We note the close match between the asymptotic power curves calculated based on (13) and the simulated curves. If we increase the number of repetitions further, the simulated power perfectly match the asymptotic one. In this example, TI outperforms the mx and the avg, in terms of power, for all false positive rates, and achieves a given power, at size α=.05, with a smaller sample size.

## 6 TRIAL APPLICATION

In this section, we first demonstrate how the two‐sample tests discussed in Section 4 can be implemented using a simple trial with hypothetical data. Then, we show the results of application to a real clinical trial.

### 6.1 Hypothetical data

Consider a simple hypothetical trial where *n* = *m* = 5 patients were assigned to each of two treatment groups, with toxicity data given in Table 1. The data were randomly generated from Poisson‐Limit models with λ=(0,5,1,5,0) and γ=(0,1,1,1.5,0) and the patients are ordered in decreasing *T*‐rank. Note that the first drug is more toxic on average, in the sense that λ≥γ coordinatewise, that is, the mean of vector of one distribution dominates the other in every coordinate. The pooled rate estimate $\hat{\lambda }$ is obtained by taking the average of each grade column in Table 1, giving $\hat{\lambda }=(0,3.3,1.1,2.9,0)$. The table also shows the TI and mx for each patient. Averaging over the TI column, we obtain mean TI values of 4.996 and 4.210 for the two groups. Hence, *S*_TI_ = 4.996 − 4.210 = 0.786. The standard error (ie, the estimated standard deviation) of this statistic is

$$\text{se}(S_{\text{TI}}):=\sqrt{\sigma_{\text{TI}}^{2}(\hat{\lambda })\left(\frac{1}{n}+\frac{1}{m}\right)}=0.251$$

using the formula in Lemma 4 to calculate $\sigma_{\text{TI}}^{2}(\hat{\lambda })=0.157$. At level α=.05, the test based on TI rejects the equality of treatments since $S_{\text{TI}}/\text{se}(S_{\text{TI}})=3.13>1.96=z_{\alpha /2}$. Similar calculations can be performed for the tests based on mx and avg, as shown in Table 2. Both tests fail to reject the equality of treatments since *S*_mx_/se(*S*_mx_) = 1.89 and *S*_avg_/se(*S*_avg_) = 1.07 are both less than $z_{\alpha /2}=1.96$.

**TABLE 1:** Frequency vector representation for the hypothetical example in Section 6.1

	Treatment	Grade 1	Grade 2	Grade 3	Grade 4	Grade 5	TI	mx	avg
Patient 1	1	0	3	0	7	0	5	4	3.40
Patient 2	1	0	5	1	6	0	5	4	3.08
Patient 3	1	0	8	1	4	0	5	4	2.69
Patient 4	1	0	4	3	3	0	4.99	4	2.90
Patient 5	1	0	4	2	3	0	4.99	4	2.89
Patient 6	2	0	1	0	3	0	4.98	4	3.50
Patient 7	2	0	1	3	1	0	4.79	4	3.00
Patient 8	2	0	4	1	1	0	4.75	4	2.50
Patient 9	2	0	2	0	1	0	4.53	4	2.67
Patient 10	2	0	1	0	0	0	2	2	2.00

**TABLE 2:** Summary measures for the hypothetical example in Section 6.1

	Treatment 1			Treatment 2								
	Mean	$M_{g}(\lambda )$	$\sigma_{g}^{2}(\lambda )$	Mean	$M_{g}(\gamma )$	$\sigma_{g}^{2}(\gamma )$	*S*_*g*_	μ	$\sigma^{2}$	se(*S*_*g*_)	$\frac{S_{g}}{\text{se}(S_{g})}$	Power
TI	4.996	4.972	0.02	4.210	4.337	1.088	0.786	0.635	1.108	0.251	3.131	0.877
mx	4	3.991	0.0142	3.6	3.634	0.698	0.4	0.357	0.7122	0.212	1.887	0.800
avg	2.992	2.9999	0.0923	2.734	3.0479	0.542	0.258	‐0.048	0.6343	0.233	1.107	0.295

Note, from Table 2, that the standard error of the two statistics *S*_TI_ and *S*_mx_ are roughly the same in this case. However, the difference in group means is much larger for TI relative to mx (|*S*_TI_|≫ |*S*_mx_|) due to patients in group 1 having many more grade 4 toxicities relative to group 2. The TI takes the frequency of grade 4 toxicities into account while the mx ignores it.

We can also use formula (13) to approximate the power of the tests. The true means are $M_{\text{TI}}(\lambda )=4.972$ and $M_{\text{TI}}(\gamma )=4.337$ using the formula from Lemma 4. Considering that the sample size is small, these values are very close to the empirical means computed from the data. The corresponding true variances are $\sigma_{\text{TI}}^{2}(\lambda )=0.02$ and $\sigma_{\text{TI}}^{2}(\gamma )=1.088$. Note how small group 1 variance is relative to group 2. The pooled normalized variance (under the alternative) is $\sigma^{2}=\sigma_{\text{TI}}^{2}(\lambda )+\rho \sigma_{\text{TI}}^{2}(\gamma )=1.108$ (since ρ=m/n=1) and the mean difference is $\mu =M_{\text{TI}}(\lambda )-M_{\text{TI}}(\gamma )=0.635$. Plugging‐in these values into (13) we obtain an approximate power of 0.877.

**FIGURE 4:**
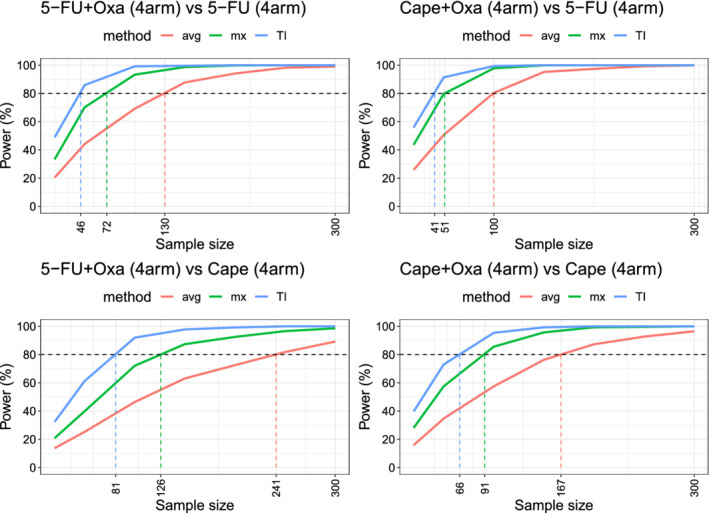
Power comparisons for detecting treatment differences in the NSABP R0‐4 clinical trial. The plots are generated based on the Poisson‐Limit models fitted to each treatment data [Colour figure can be viewed at wileyonlinelibrary.com]

Similarly, for the mx, note how close the true means are to the empirical ones (cf Table 2). The fact that for treatment 1, $\lambda_{4}$ is quite large—hence most patients show at least one grade 4 toxicity—is reflected in the small value of the variance $\sigma_{\text{mx}}^{2}(\lambda )$. That is, for the majority of patients in group 1, mx will be 4, hence its mean will be concentrated near 4. In this case, we have $\sigma^{2}=0.712$ and μ=0.357 giving an approximate power of 0.800.

For the avg, group 1 variance is higher than both TI and mx, though still quite small relative to group 2. The mean difference is very small in this case, μ=−0.048. The negative sign indicates that treatment 1 is less toxic on average than treatment 2 (in expectation), which is quite counter‐intuitive since treatment 1 is more toxic in terms of the extreme grades. Even more surprisingly, we have $M_{\text{avg}}(\lambda )<M_{\text{avg}}(\gamma )$ despite λ≥γ coordinatewise. That is, the frequency of all the grades are higher on average for treatment 1, but its expected avg is lower. This can be confirmed empirically, for large *n*, by observing that *S*_avg_ turns out to be negative most of the time. It can be explained by noting that $\lambda_{2}$ being much larger than $\gamma_{2}$ biases the avg for group 1 toward lower grades. This shows a clear deficiency of the avg in ordering toxicities. Plugging‐in μ=−0.048 and $\sigma^{2}=0.635$ into (13), we obtain an approximate power of 0.295.

### 6.2 Application to clinical trial data

We use the data from the NSABP R0‐4 rectal cancer clinical trial as a case study for comparing the power of the three summary statistics (TI, mx, and avg) in detecting treatment differences. NSABP R0‐4 was a phase 3 trial conducted between July 2004 and August 2013 (NCT00058474). Eligible patients were diagnosed with surgically resectable stage II or III rectal adenocarcinoma. Patients were assigned to four different treatments: (1) infusional 5‐fluorouracil (5FU); (2) oral capecitabine (CAPE); (3) combination of 5FU and oxaliplatin (OX); (4) combination of CAPE and Ox. The trial included 1608 participants. From these, 50 patients were ineligible or had missing data. The analytic sample consisted of 1558 individuals. CONSORT diagram is available in Reference 21. Main study description is available in Reference 23.

We use the power analysis to compare the performance of the three methods in detecting treatment differences. We first fit a Poisson‐Limit model (see (5)) to each treatment data which amounts to estimating its rate vector using the sample mean of the corresponding RFVs; see for example (11), but applied only to a single sample. Once the model for each treatment is specified, we can compare the methods based on their power in differentiating pairs of treatments. For each method, we plot the power vs the sample size. These plots can be obtained by simulating from the fitted models (a form of parametric bootstrap) or by using the asymptotic power formula (13) which, as was shown in the previous section, gives accurate results even for small sample sizes.

Figure 4 shows the power plots for testing four pairs of treatments that are known to be different. All tests are performed at 5% significance level. The plots show that TI has a greater power in detecting differences between treatments in all the four cases. That is, using TI, the required number of patients to detect treatment differences is smaller. We note that Reference 21 performs a similar power analysis on the same dataset. They provide no model for the data and instead use bootstrap to perform the power analysis. The tests they consider are based on Wilcoxson signed‐rank statistic. In contrast, we provide a model for the data and use parametric bootstrap (or exact asymptotic power curves) to perform the power analysis. Our test statistics are also different and based on the difference‐in‐mean statistics. Compared with the results of Reference 21, Figure 4 shows that the test statistics considered here achieve a given power with a smaller sample size. We observe a similar relative ranking of TI, average and max‐grade as that observed by Gresham et al.^21^

## 7 DISCUSSION

In this article, we studied the mathematical and statistical properties of the toxicity index. We showed that the TI can be stated solely as a function of the reduced frequency vector, with a tractable closed‐form formula. We introduced T‐rank preservation as a desirable property that allows investigators to achieve clinically‐meaningful ranking of the toxicity profiles. We showed that compared with competing summary measures (ie, the max‐grade and the average‐grade), TI is the only measure that preserves the T‐rank and is an injective (ie, one‐to‐one) mapping on the space of toxicity frequencies. Neither max‐grade, nor the average‐grade are injective or T‐rank preserving. The max‐grade loses information in the toxicity profile by only looking at the highest grade. The average‐grade loses the information about the extreme toxicities by equally weighting low and high grades.

To statistically compare various toxicity measures, we proposed a Poisson‐Limit model for modeling sparse toxicity data via their reduced (ie, 0‐removed) frequency vectors. Under this model, we developed a general two‐sample test for detecting differences among two population of toxicity data. We derived formulas for the asymptotic power of the test, for the three toxicity measures (TI, max‐grade, and average‐grade) and calculated their asymptotic relative efficiencies (AREs). We empirically demonstrated that TI has a higher ARE that the other two summary measures, with high probability, under a random Poisson‐Limit model. We also illustrated that TI achieves a higher power in detecting treatment differences in a cancer trial, validating the empirical results of Reference 21. The framework we developed in this article allows investigators to compare toxicity‐summary methods in any clinical trial, and to analytically evaluate new proposals for toxicity summaries.

The TI can be generalized to accommodate noninteger toxicity grades if the clinician believes that a toxicity has a different impact than the observed grade.^11^, ^25^ Furthermore, the TI can be used in any risk assessment application with a grade system similar to CTCAE, provided that the grades for different inputs (eg, items) are equivalent. In Reference 26, the TI was used to score surgical complications based on the Clavien‐Dindo system.^27^ More broadly, the analysis in this article applies to any application where integer scores on multiple events are recorded for a collection of subjects, assuming that the resulting score array is sparse. We are currently investigating the benefits of using TI for patient reported outcomes (PROs) data,^28^ extending the TI for multiple cycles and searching for other T‐rank preserving summary measures.

## 1

### A.1 Proof of Equation (5)

Recall that *X*_∗_ = (*X*_0_, *X*_1_ … , *X*_*d*_) ∼ Mult(*d*, *p*_∗_) where *p*_∗_ = (*p*_0_, *p*_1_ … , *p*_*K*_). It is well known that the moment‐generating function (MGF) of *X*_∗_ is given by $𝔼\left[\exp\left(\sum_{i=0}^{d}t_{i}X_{i}\right)\right]={\left(\sum_{i=0}^{d}p_{i}e^{t_{i}}\right)}^{d}$. Setting *t*_0_ = 0 and noting that $p_{0}=1-\sum_{i=1}^{K}p_{i}$ and $p_{i}=\lambda_{i}/d$ for *i* = 1, … , *K*, we obtain the MGF of *X*,

$$M_{X}(t)=𝔼\left[\exp\left(\overset{d}{\underset{i=1}{\sum }}t_{i}X_{i}\right)\right]={\left(1+\frac{1}{d}\overset{d}{\underset{i=1}{\sum }}\lambda_{i}(e^{t_{i}}-1)\right)}^{d}.$$

We have

$$M_{X}(t)\to \exp\left(\overset{d}{\underset{i=1}{\sum }}\lambda_{i}(e^{t_{i}}-1)\right)=\overset{d}{\underset{i=1}{\prod }}m(t_{i};\lambda_{i}),\;\text{as}\;d\to \infty ,$$

where $m(x;\mu )=e^{\lambda (e^{x}-1)}$ is the MGF of a Poisson random variable with mean μ. The proof is complete.

### A.2 Proofs of Section 3

Proof of Proposition 1 Assume that *Y*_*i*_ = *k* for *r*_*k*_ ≤ *i* ≤ *r*_*k* − 1_ for *k* = 1, … , *K* with 1 = *r*_*K*_ ≤ ⋯ ≤ *r*_1_ ≤ *r*_0_ = *n*. Note that *x*_*k*_ = *r*_*k* − 1_ − *r*_*k*_ + 1. For *i* ∈ ⟦*r*_*k*_, *r*_*k* − 1_⟧, we have

$$w_{i}(Y):=\overset{i-1}{\underset{j=1}{\prod }}(1+Y_{j})={\left(1+k\right)}^{i-r_{k}}\overset{K}{\underset{\lambda =k+1}{\prod }}{\left(1+\lambda \right)}^{x_{\lambda }}=\frac{{\left(1+k\right)}^{i-r_{k}}}{g_{k+1}(x)}.$$

Then,

$$\begin{array}{llll} \tau_{k}(Y)-\tau_{k-1}(Y) & =\overset{n}{\underset{i=1}{\sum }}\frac{Y_{i}}{w_{i}(Y)}1\{Y_{i}=k\}\; & & \\ & =\underset{i\in ⟦r_{k},r_{k-1}⟧}{\sum }\frac{kg_{k+1}(x)}{{\left(1+k\right)}^{i-r_{k}}}\; & & \\ & =k\;g_{k+1}(x)\underset{i\in ⟦r_{k},r_{k-1}⟧}{\sum }{\left(1+k\right)}^{-(i-r_{k})}\; & & \\ & =(k\;g_{k+1}(x))\frac{1-{\left(1+k\right)}^{r_{k-1}-r_{k}+1}}{1-{\left(1+k\right)}^{-1}}=(k+1)\;g_{k+1}(x)[1-{\left(1+k\right)}^{-x_{k}}]\; & & \end{array}$$

which is the desired result.

Proof of Lemma 1 Assume *x* ≻ *y* and *y* ≻ *z*. By assumption, *x* ≠ *y* and *y* ≠ *z*. We consider three cases:

(i) *i*(*x*, *y*) = *i*(*y*, *z*) =  : *ℓ*. Then, it is not hard to argue that *i*(*x*, *z*) = *ℓ* and since *x*_*ℓ*_ > *y*_*ℓ*_ > *z*_*ℓ*_, we have *x* ≻ *z*.
(ii) *ℓ*_1_: = *i*(*x*, *y*) > *i*(*y*, *z*). Then, *i*(*x*, *z*) = *ℓ*_1_ and $x_{\ell_{1}}>y_{\ell_{1}}=z_{\ell_{1}}$. Hence, *x* ≻ *z*.
(iii) *i*(*x*, *y*) < *i*(*y*, *z*) =  : *ℓ*_0_. Then, *i*(*x*, *z*) = *ℓ*_0_ and $x_{\ell_{0}}=y_{\ell_{0}}>z_{\ell_{0}}$. Hence, *x* ≻ *z*.

The proof is complete.

Proof of Theorem 1 Assume *K* ≥ 2. Consider the following two vectors

$$\left\{\begin{array}{cc} x_{K}=m\\ x_{j}=0\; & j\neq K \end{array}\right.,\;\left\{\begin{array}{cc} y_{K}=m-1\\ y_{j}=\infty \; & j<K \end{array}\right..$$

If is enough to show that TI(*x*) ≥ TI(*y*). In fact, we show equality. To simplify, let us write *u* = (1 + *K*)^−*m*^. Then,

$$\text{TI}(x)=g_{K+1}(x)(K+1)\left(1-{\left(K+1\right)}^{-m}\right)=(K+1)(1-u).$$

Note that all the lower terms are zero in the expansion. On the other hand, we have $g_{K}(y)={\left(1+K\right)}^{y_{K}}={\left(1+K\right)}^{m-1}$ and *g*_*k*_(*y*) = *∞* for *k* < *K*. It follows that

$$\begin{array}{llll} \text{TI}(y) & =g_{K+1}(y)(K+1)\left(1-{\left(K+1\right)}^{-m+1}\right)+K\;g_{K}(y)\; & & \\ & =(K+1)\left(1-{\left(K+1\right)}^{-m+1}\right)+K{\left(K+1\right)}^{-m+1}\; & & \\ & =(K+1)[1-(K+1)u]+K(K+1)u.\; & & \end{array}$$

Then,

TI(x)−TI(y)=(K+1)[(K+1)u−u]−K(K+1)u=0.

The proof for the general case follows by considering the largest grade on which *x* and *y* differ, which without loss of generality (WLOG) we assume to be *K*. Assume WLOG that *x*_*K*_ > *y*_*K*_. Then, setting *x*_*j*_ = 0 for all *j* ≠ *K* only decreases TI(*x*). Similarly, increasing *y*_*K*_ to *x*_*K*_ − 1 and increasing all *y*_*j*_ = *∞*, only increases TI(*y*). We have shown that even in this worst case TI(*x*) ≥ TI(*y*). So all the other cases follow.

### A.3 Proofs of Section 4

Proof of Theorem 2 For simplicity, and without loss of generality, assume that m=n/ρ. Let $T_{g}(X)=\frac{1}{n}\sum_{i=1}^{n}g(X^{\left(i\right)})$ and $T_{g}(Y)=\frac{1}{m}\sum_{i=1}^{m}g(Y^{\left(i\right)})$ so that $S_{g}=T_{g}(X)-T_{g}(Y)$. Then, by the central limit theorem

$$\begin{array}{llll} \sqrt{n}\left[T_{g}(X)-M_{g}(\lambda )\right] & ⇝N(0,\sigma_{g}^{2}(\lambda )),\; & & \\ \sqrt{n}\left[T_{g}(Y)-M_{g}(\gamma )\right] & ⇝N(0,\rho \sigma_{g}^{2}(\gamma )).\; & & \end{array}$$

Subtracting, we get

$$\sqrt{n}\left(S_{g}+[M_{g}(\gamma )-M_{g}(\lambda )]\right)⇝N(0,\sigma_{g}^{2}(\lambda )+\rho \sigma_{g}^{2}(\gamma )).$$

Under the null distribution, $M_{g}(\lambda )-M_{g}(\gamma )=0$, and the test that rejects when

$$|S_{g}|>z_{\alpha /2}\sqrt{\frac{\sigma_{g}^{2}(\lambda )(1+\rho )}{n}}$$

has asymptotic level α. Since $\hat{\lambda }\to \lambda$ and $\sigma_{g}^{2}(\cdot )$ is continuous, by the continuous mapping theorem, the same result holds with λ replaced with $\hat{\lambda }$. Let $\mu =M_{g}(\gamma )-M_{g}(\lambda )$, $\tau =z_{\alpha /2}\sigma_{g}(\lambda )\sqrt{1+\rho }$ and $\sigma^{2}=\sigma_{g}^{2}(\lambda )+\rho \sigma_{g}^{2}(\gamma )$. It follows that for large *n*, the power is close to

(A1) $$\begin{array}{llll} \text{power} & ={\mathbb{P}}_{1}\left(\frac{\sqrt{n}(S_{g}+\mu )}{\sigma }>\frac{\tau +\sqrt{n}\mu }{\sigma }\right)+{\mathbb{P}}_{1}\left(\frac{-\sqrt{n}(S_{g}+\mu )}{\sigma }>\frac{\tau -\sqrt{n}\mu }{\sigma }\right)\; & & \\ & \approx Q\left(\frac{\tau +\sqrt{n}\mu }{\sigma }\right)+Q\left(\frac{\tau -\sqrt{n}\mu }{\sigma }\right). \end{array}$$

More precisely, if μ=0, then the power of the test converges to 2Q(τ/σ) which is the desired result for part (a). If in addition $\sigma_{g}^{2}(\lambda )=\sigma_{g}^{2}(\gamma )$, then $\tau /\sigma =z_{\alpha /2}$, hence the power converges to $2Q(z_{\alpha /2})=\alpha$. For part (c), we note that by the Taylor expansion $\sqrt{n}[M_{g}(\gamma )-M_{g}(\lambda )]=\langle \nabla M_{g}(\lambda ),\delta \rangle +O(n^{-1/2})$, and $\sigma_{g}^{2}(\gamma )\to \sigma_{g}^{2}(\lambda )$ by the continuity of $\sigma_{g}^{2}(\cdot )$. Hence, $\tau /\sigma \to z_{\alpha /2}$, $\sigma \to \sigma_{g}(\lambda )\sqrt{1+\rho }$ and $\sqrt{n}\mu \to \langle \nabla M_{g}(\lambda ),\delta \rangle$. The result then follows by considering the asymptotic expression (A1).

Proof of Lemma 2 Under the Poisson model, $X|X_{+}=m∼\text{Mult}(m,\bar{\lambda })$ where $\bar{\lambda }=\lambda /\lambda_{+}$. Assuming *m* ≠ 0, we have

$${𝔼}_{\lambda }(\text{avg}(X)|X_{+}=m)=\frac{1}{m}\underset{r}{\sum }r𝔼[X_{r}|X_{+}=m]=\frac{1}{m}\underset{r}{\sum }rm{\bar{\lambda }}_{r}=m_{1}(\bar{\lambda }).$$

We obtain ${𝔼}_{\lambda }[\text{avg}(X)|X_{+}]=m_{1}(\bar{\lambda })\cdot 1\{X_{+}>0\}$. Since $X_{+}∼\text{Poi}(\lambda_{+})$, by smoothing,

$${𝔼}_{\lambda }[\text{avg}(X)]=m_{1}(\bar{\lambda })\;\mathbb{P}(X_{+}>0)=m_{1}(\bar{\lambda })(1-e^{-\lambda_{+}}),$$

which is the desired expression. Now, by the law of total variance

$$\sigma_{\mathrm{var}}^{2}(\lambda )=𝔼\left[\mathrm{var}\left(\text{avg}(X)|X_{+}\right)\right]+\mathrm{var}\left(𝔼[\text{avg}(X)|X_{+}]\right).$$

We have $\mathrm{var}(𝔼[\text{avg}(X)|X_{+}])=m_{1}^{2}(\bar{\lambda })\mathrm{var}(1\{X_{+}>0\})=m_{1}^{2}(\bar{\lambda })p(1-p)$ where $p=1-e^{-\lambda_{+}}$. On the other hand, assuming *m* ≠ 0, conditioned on *X*_+_ = *m*, we can represent $X_{r}=\sum_{j=1}^{m}W^{\left(j\right)}$ where $W^{\left(j\right)}∼\text{Mult}(1,\bar{\lambda })$ drawn i.i.d. Note that $\sum_{r}rW^{\left(j\right)}$ is categorical variable taking values 1, … , *K* with probabilities ${\bar{\lambda }}_{1},\ldots ,{\bar{\lambda }}_{K}$. Hence,

$$\begin{array}{llll} \mathrm{var}\left(\text{avg}(X)|X_{+}=m\right) & =\frac{1}{m^{2}}\mathrm{var}(\underset{r}{\sum }rX_{r})\; & & \\ & =\frac{1}{m^{2}}\mathrm{var}\left(\underset{j}{\sum }\underset{r}{\sum }rW_{r}^{\left(j\right)}\right)=\frac{1}{m}\mathrm{var}\left(\underset{r}{\sum }rW_{r}^{\left(1\right)}\right)=\frac{m_{2}(\bar{\lambda })}{m},\; & & \end{array}$$

where $m_{2}(\bar{\lambda })=\sum_{r}r^{2}{\bar{\lambda }}_{r}-{\left(\sum_{r}r{\bar{\lambda }}_{r}\right)}^{2}$ is the variance of $\sum_{r}rW^{\left(1\right)}$. Hence,

$${𝔼}_{\lambda }\left[{\mathrm{var}}_{\lambda }\left(\text{avg}(X)|X_{+}\right)\right]={𝔼}_{\lambda }\left(\frac{1\{X_{+}>0\}}{X_{+}}\right)m_{2}(\bar{\lambda }).$$

Since $X_{+}∼\text{Poi}(\lambda_{+})$, we have (cf Reference 29)

$${𝔼}_{\lambda }\left(\frac{1\{X_{+}>0\}}{X_{+}}\right)=e^{-\lambda_{+}}\overset{\infty }{\underset{k=1}{\sum }}\frac{\lambda_{+}^{k}}{k\cdot k!}=e^{-\lambda_{+}}\text{Er}(\lambda_{+}).$$

The proof is complete.

Proof of Lemma 3 Let *B*_*r*_ = 1{*X*_*r*_ > 0} and $p_{r}=\mathbb{P}(X_{r}>0)=1-e^{-\lambda_{r}}$. Then, $\text{mx}(X)=\max\{r:B_{r}=1\}$ and we have

$$\mathbb{P}(\text{mx}(X)=k)=p_{k}\overset{K}{\underset{r=k+1}{\prod }}(1-p_{r})=e^{-W_{k+1}(\lambda )}-e^{-W_{k}(\lambda )},$$

if *k* ≠ 0 which gives the desired result.

Proof of Lemma 4 Assume that X∼Poi(λ) and let *g*_*k*_ = *g*_*k*_(*X*). Then,

$$𝔼[f(X)]=\overset{K+1}{\underset{k=2}{\sum }}k\left(𝔼[g_{k}]-𝔼[g_{k-1}]\right).$$

Recall that if N∼Poi(λ), then $𝔼[z^{N}]=e^{\lambda (z-1)}$. Thus,

$$𝔼\left[{\left(1+i\right)}^{-X_{i}}\right]=\exp\left[\lambda_{i}({\left(1+i\right)}^{-1}-1)\right]=e^{-a_{i}\lambda_{i}}.$$

By the independence of the coordinates, $𝔼[g_{k}]=\prod_{i=k}^{K}𝔼[{\left(1+i\right)}^{-X_{i}}]=e^{-U_{k}(\lambda )}$. Equation (18) follows. For the variance, we note that

$$\begin{array}{llll} 𝔼[g_{k}g_{\ell }] & =𝔼\left[\overset{K}{\underset{i=k}{\prod }}{\left(1+i\right)}^{-X_{i}}\overset{K}{\underset{j=\ell }{\prod }}{\left(1+j\right)}^{-X_{j}}\right]\; & & \\ & =𝔼\left[\overset{k\vee \ell -1}{\underset{i=k\wedge \ell }{\prod }}{\left(1+i\right)}^{-X_{i}}\overset{K}{\underset{i=k\vee \ell }{\prod }}{\left(1+i\right)}^{-2X_{i}}\right]=e^{-V_{k,\ell }(\lambda )},\; & & \end{array}$$

where the final equality is by independence and the definition of $V_{k,\ell }(\lambda )$. We have

$$\mathrm{var}(f(X))=\underset{k}{\sum }\underset{\ell }{\sum }k\ell \text{cov}\left(g_{k}-g_{k-1},g_{\ell }-g_{\ell -1}\right).$$

Noting that $\text{cov}(g_{k},g_{\ell })=\Gamma_{k,\ell }(\lambda )$, Equation (19) follows.

We have

$$\xi (z)=\overset{K}{\underset{k=1}{\sum }}\overset{K}{\underset{i=1}{\sum }}1\{i\leq k\}\left[e^{-z_{k+1}}-e^{-z_{k}}\right]=\overset{K}{\underset{i=1}{\sum }}\overset{K}{\underset{k=i}{\sum }}\left[e^{-z_{k+1}}-e^{-z_{k}}\right]=\overset{K}{\underset{i=1}{\sum }}\left[e^{-z_{K+1}}-e^{-z_{i}}\right].$$

Recalling that *z*_*K* + 1_ = 1 finishes the proof.

## References

[1] Miller AB , Hoogstraten BFAU , Staquet MFAU , Winkler A . Reporting results of cancer treatment. Cancer. 1981;47(1):207‐214. 7459811 10.1002/1097-0142(19810101)47:1<207::aid-cncr2820470134>3.0.co;2-6

[2] Trotti A , Colevas AD , Setser A , Basch E . Patient‐reported outcomes and the evolution of adverse event reporting in oncology. J Clin Oncol. 2007;25(32):5121‐5127. 17991931 10.1200/JCO.2007.12.4784

[3] CTCAE Common Terminology Criteria for Adverse Events (CTCAE), Version 5.

[4] Trotti A , Colevas AD , Setser A , et al. CTCAE v3.0: development of a comprehensive grading system for the adverse effects of cancer treatment. Seminars in Radiation Oncology. 2003;13(3):176‐181. 12903007 10.1016/S1053-4296(03)00031-6

[5] Thanarajasingam G , Hubbard JM , Sloan JA , Grothey A . The imperative for a new approach to toxicity analysis in oncology clinical trials. J National Cancer Inst. 2015;107(10):djv216. 10.1093/jnci/djv216 26232762

[6] Singer DS , Jacks T , Jaffee E . A US “Cancer Moonshot” to accelerate cancer research. Science. 2016;353(6304):1105‐1106. 27605537 10.1126/science.aai7862

[7] Forastiere AA , Goepfert H , Maor M , et al. Concurrent chemotherapy and radiotherapy for organ preservation in advanced laryngeal cancer. N Engl J Med. 2003;349(22):2091‐2098. 14645636 10.1056/NEJMoa031317

[8] Adelstein DJ , Li Y , Adams GL , et al. An intergroup phase III comparison of standard radiation therapy and two schedules of concurrent chemoradiotherapy in patients with unresectable squamous cell head and neck cancer. J Clin Oncol. 2003;21(1):92‐98. 12506176 10.1200/JCO.2003.01.008

[9] Baselga J , Trigo JM , Bourhis J , et al. Phase II multicenter study of the antiepidermal growth factor receptor monoclonal antibody cetuximab in combination with platinum‐based chemotherapy in patients with platinum‐refractory metastatic and/or recurrent squamous cell carcinoma of the head and neck. J Clin Oncol. 2005;23(24):5568‐5577. 16009950 10.1200/JCO.2005.07.119

[10] Trotti A , Pajak TF , Gwede CK , et al. TAME: development of a new method for summarising adverse events of cancer treatment by the radiation therapy oncology group. Lancet Oncol. 2007;8(7):613‐624. 17543584 10.1016/S1470-2045(07)70144-4

[11] Lee SM , Hershman DL , Martin P , Leonard JP , Cheung YK . Toxicity burden score: a novel approach to summarize multiple toxic effects. Ann Oncol. 2011;23(2):537‐541. 21536663 10.1093/annonc/mdr146 PMC3295018

[12] Thanarajasingam G , Atherton PJ , Novotny PJ , Loprinzi CL , Sloan JA , Grothey A . Longitudinal adverse event assessment in oncology clinical trials: the Toxicity over Time (ToxT) analysis of alliance trials NCCTG N9741 and 979254. Lancet Oncol. 2016;17(5):663‐670. 27083333 10.1016/S1470-2045(16)00038-3 PMC4910515

[13] Gordon NH , Willson JK . Using toxicity grades in the design and analysis of cancer phase I clinical trials. Stat Med. 1992;11(16):2063‐2075. 1293668 10.1002/sim.4780111604

[14] Wang C , Chen TT , Tyan I . Designs for phase I cancer clinical trials with differentiation of graded toxicity. Commun Stat. 2000;29(5‐6):975‐987.

[15] Bekele BN , Thall PF . Dose‐finding based on multiple toxicities in a soft tissue sarcoma trial. J Am Stat Assoc. 2004;99(465):26‐35.

[16] Van Meter EM , Garrett‐Mayer E , Bandyopadhyay D . Proportional odds model for dosefinding clinical trial designs with ordinal toxicity grading. Stat Med. 2011;30(17):2070‐2080. 21344472 10.1002/sim.4069 PMC3117067

[17] Chen Z , Tighiouart M , Kowalski J . Dose escalation with overdose control using a quasi‐continuous toxicity score in cancer phase I clinical trials. Contemp Clin Trials. 2012;33(5):949‐958. 22561391 10.1016/j.cct.2012.04.007 PMC4046335

[18] Tighiouart M , Cook‐Wiens G , Rogatko A . Escalation with overdose control using ordinal toxicity grades for cancer phase I clinical trials. J Probab Stat. 2012;2012:1‐18.

[19] Gelber RD , Goldhirsch A , Cole BF , Wieand HS , Schroeder G , Krook JE . A quality‐adjusted time without symptoms or toxicity (Q‐TWiST) analysis of adjuvant radiation therapy and chemotherapy for resectable rectal cancer. J Natl Cancer Inst. 1996;88(15):1039‐1045. 8683634 10.1093/jnci/88.15.1039

[20] Rogatko A , Babb JS , Wang H , Slifker MJ , Hudes GR . Patient characteristics compete with dose as predictors of acute treatment toxicity in early phase clinical trials. Clin Cancer Res. 2004;10(14):4645‐4651. 15269136 10.1158/1078-0432.CCR-03-0535

[21] Gresham G , Diniz MA , Razaee ZS , et al. Evaluating treatment tolerability in cancer clinical trials using the toxicity index. JNCI J National Cancer Inst. 2020;112(12):1266‐1274. 10.1093/jnci/djaa028 PMC7735773 32091598

[22] Russell MM , Ganz PA , Lopa S , et al. Comparative effectiveness of sphincter‐sparing surgery versus abdominoperineal resection in rectal cancer: patient‐reported outcomes in national surgical adjuvant breast and bowel project randomized trial R‐04. Ann Surg. 2015;261(1):144. 24670844 10.1097/SLA.0000000000000594 PMC4379325

[23] Allegra CJ , Yothers G , O'Connell MJ , et al. Neoadjuvant 5‐FU or capecitabine plus radiation with or without oxaliplatin in rectal cancer patients: a phase III randomized clinical trial. J National Cancer Inst. 2015;107(11):djv248. 10.1093/jnci/djv248 PMC4849360 26374429

[24] Vaart AW . Asymptotic Statistics. Cambridge, MA: Cambridge University Press; 2000.

[25] Yuan Z , Chappell R , Bailey H . The continual reassessment method for multiple toxicity grades: a Bayesian quasi‐likelihood approach. Biometrics. 2007;63(1):173‐179. 17447942 10.1111/j.1541-0420.2006.00666.x

[26] Anger JT , Mueller ER , Tarnay C , et al. Robotic compared with laparoscopic sacrocolpopexy: a randomized controlled trial. Obstet Gynecol. 2014;123(1):5. 24463657 10.1097/AOG.0000000000000006 PMC4266590

[27] Dindo D , Demartines N , Clavien P‐A . Classification of surgical complications: a new proposal with evaluation in a cohort of 6336 patients and results of a survey. Ann Surg. 2004;240(2):205. 15273542 10.1097/01.sla.0000133083.54934.ae PMC1360123

[28] Nayfield SG , Ganz PA , Moinpour CM , Cella DF , Hailey BJ . Report from a National Cancer Institute (USA) workshop on quality of life assessment in cancer clinical trials. Qual Life Res. 1992;1(3):203‐210. 1363776 10.1007/BF00635619

[29] Audenaert KMR . Inverse moments of univariate discrete distributions via the Poisson expansion; 2008. arXiv preprint arXiv:0809.4155.

